# Paraoxonase 1 and Chronic Kidney Disease: A Meta-Analysis

**DOI:** 10.3390/jcm12031199

**Published:** 2023-02-02

**Authors:** Jun Watanabe, Kazuhiko Kotani, Alejandro Gugliucci

**Affiliations:** 1 Division of Community and Family Medicine, Jichi Medical University, 3311-1 Yakushiji, Shimotsuke-City 329-0498, Japan; 2 Glycation, Oxidation and Disease Laboratory, Touro University-California, Vallejo, CA 94592, USA

**Keywords:** antioxidants, arylesterase, cardiovascular disease, kidney failure, paraoxonase, reactive oxygen species

## Abstract

Oxidative stress is known to be associated with the pathophysiology of chronic kidney disease (CKD). Paraoxonase 1 (PON1) is an antioxidant enzyme that has been proposed as a biomarker for CKD. While several studies have reported an association between serum PON1 activity and CKD, consensus based on systematically analyzed data remains necessary. We set out to conduct a meta-analysis of literature on PON1 in CKD. Electronic databases, such as MEDLINE, Embase and CENTRAL, were searched for available studies on PON1 activity in patients with CKD (without dialysis) as published before December 2022. A random-effects meta-analysis was performed. In total, 24 studies (22 studies on paraoxonase and 11 on arylesterase activity) were eligibly identified. Patients with CKD showed a lower activity of paraoxonase (standard mean difference [SMD], −1.72; 95% confidence interval [CI], −2.15 to −1.29) and arylesterase (SMD, −2.60; 95%CI, −3.96 to −1.24) than healthy controls. In the subgroup analyses, paraoxonase activity was lower in chronic kidney failure (CKF), an advanced stage of CKD, than in non-CKF. In summary, PON1 activity is low in patients with CKD, suggesting that the antioxidant defense by PON1 is impaired in CKD. The decrease in enzyme activity is pronounced in advanced CKD showing some variability depending on the substrate employed to measure PON1 activity. Further studies are warranted.

## 1. Introduction

Chronic kidney disease (CKD) involves a loss of kidney function leading to a sociomedical burden associated with low quality of health and high economic cost and is also a leading cause of cardiovascular mortality [1,2,3]. The global prevalence of CKD is generally estimated as high as 14% [4,5]. As CKD can ultimately result in end-stage kidney failure (ESRD) with renal replacement treatment such as dialysis, the preventative measures for CKD to retard ESRD and dialysis are a great issue worldwide.

Patients with CKD have multiple cardiometabolic disorders including hypertension, insulin resistance, diabetes and dyslipoproteinemias, along with other CKD-related physical abnormalities, which can produce oxidative stress [6,7]. Patients with CKD frequently have dyslipoproteinemias, such as low levels of high-density lipoprotein (HDL) and high levels of triglycerides [8,9]. Low HDL-cholesterol levels are well known to be an atherosclerotic risk index, although in reality, it is dysfunctional HDL that may be implicated as a contributing factor in atherogenesis. Indeed, HDL not only plays a role in reverse cholesterol transport from peripheral cells and more so in reverse remnant cholesterol transport (called RRCT) but also inhibits oxidation of low-density lipoprotein (LDL) that contributes to atherogenesis [8,10,11,12]. Thus, there is a need to control oxidative stress to prevent the development of atherosclerosis in CKD. 

The essential mechanism by which HDL inhibits oxidation of LDL is shown to be partially enzymatic [13]. Paraoxonase 1 (PON1) is getting a lot of attention in the physiology of the atheroprotective function of HDL [11,13]. PON1 is composed of 354 amino-acids with a molecular weight of 43 kDa and is encoded by the PON1 gene [14]. PON1 is a promiscuous esterase whose physiological function is believed to be that of a lactonase but its activity is conveniently assessed in the laboratory as an esterase, using an array of substrates. Most studies employ phenylacetate and show the results as arylesterase or paraoxon reporting the activity as paraoxonase [15]. In fact, the significance of PON1 activities, as arylesterase or paraoxonase, has been investigated beyond atherosclerosis and CVD, including lung diseases, diabetes, and neurological pathologies [16,17,18,19]. 

The relationship between PON1 and CKD is also of interest [20,21,22], given on the one hand the accelerated atherogenesis that is a characteristic of this entity and on the other hand the known impairment of HDL metabolism in CKD associated with increased oxidative stress. However, data on the association between PON1 and CKD in the literature have shown mixed findings and have not been fully summarized using systematically-analyzed methods. Therefore, the present study aimed to summarize current evidence on PON1 activity in patients with CKD (without dialysis) via a systematic meta-analysis of available clinical studies. 

## 2. Materials and Methods

The present study was reported according to the statement of Preferred Reporting Items for Systematic Reviews and Meta-Analyses, PRISMA [23]. The protocol was registered in PROSPERO (CRD42023389430).

The study was performed using electronic search engines on MEDLINE (1946 to the present), Embase (1974 to the present) and CENTRAL (from inception to the present) to identify published literature based on the combined keywords of aryldialkyphosphatase and chronic kidney disease until 4 December 2022 (Appendix A). The inclusion criteria were as follows: (1) prospective and retrospective cohort studies and case-control studies; and (2) articles that evaluated the PON1 activity in patients with CKD and without dialysis. The exclusion criteria were as follows: (1) case reports, case series, reviews, and meta-analysis; (2) patients aged under 18 years; and (c) articles with undetectable data. The primary outcomes were the levels of paraoxonase and/or arylesterase activity in CKD. 

The reviewers independently reviewed abstracts and titles identified by the searches. After the title and abstract screening, the full text was reviewed to meet the review criteria. Data extraction was carried out from each study in terms of author name, year of publication, country, number of patients, age, and outcomes. The risk of bias was evaluated using an 11-item checklist which was recommended by Agency for Healthcare Research and Quality (AHRQ) [24]. 

Heterogeneity was assessed by a visual inspection of forest plots and using the I^2^ statistic (I^2^ values of 0–40%: it might not be important; 30–60%: it may represent a moderate heterogeneity; 50–90%: it may represent a substantial heterogeneity; 75–100%: it may represent a considerable heterogeneity) [25]. When there was a substantial heterogeneity (I^2^ > 50%), the reasons for heterogeneity were evaluated using subgroup analyses by CKD stages (estimated glomerular filtration rate eGFR ≥ 30 or eGFR < 30 as defined as chronic kidney failure CKF) based on the international guidelines [6], diabetes status (patients with or without diabetes), and region (Asia, Western countries, or others). Renal function was estimated using serum creatinine levels, mean age and body mass index of patients. We performed sensitivity analyses excluding the studies that treated all cases with abnormal eGFR levels as CKD. The publication bias was assessed by a visual inspection of the funnel plot [25]. The standardized mean difference (SMD) with a 95% confidence interval (CI) in the PON1 levels in patients with CKD was analyzed. A random-effects meta-analysis was performed using RevMan version 5.4.1 (The Cochrane Collaboration, Copenhagen, Denmark). 

## 3. Results

**Figure 1:**
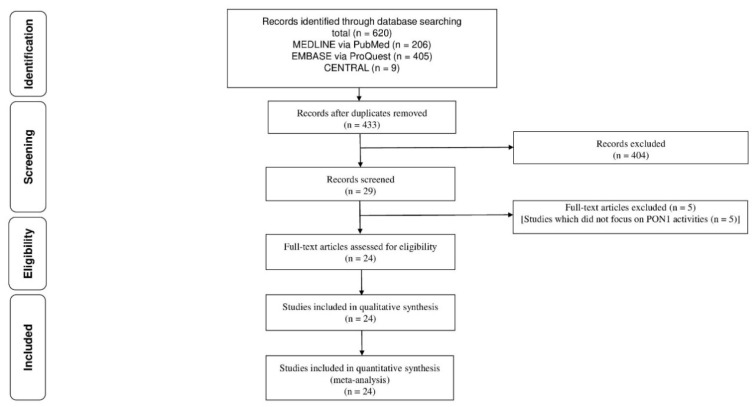
Flow diagram of selection of articles that reported the PON1 in patients with CKD.

Figure 1 shows the flow diagram to select the articles on PON1 in patients with CKD. After removing duplicated records, our initial search identified 433 records. A review of the title and abstract reduced the number to 29 studies that were read in full. Based on review of the full text of articles, five studies were excluded as they did not focus on PON1 activity. Finally, a total of 24 studies were identified that met inclusion and exclusion criteria [26,27,28,29,30,31,32,33,34,35,36,37,38,39,40,41,42,43,44,45,46,47,48,49]. 

Table 1 lists the characteristics of those eligible studies. These studies were based on case-control or cross-sectional study design. Of 24 studies, 22 reported on paraoxonase activity [26,27,28,29,30,31,32,33,34,35,36,37,38,39,40,41,42,44,46,47,48,49] and 11 on arylesterase activity [28,30,32,34,39,41,42,43,45,46,47]. In the studies that were restricted to patients with CKF (defined as eGFR < 30 [6]), 10 reported on paraoxonase activity [28,29,30,31,32,35,38,48,49] and 3 on arylesterase activity [28,30,32]. The overall risks of bias were median 7, range 3–9, using an 11-item checklist by AHRQ (Appendix B). 

**Figure 2:**
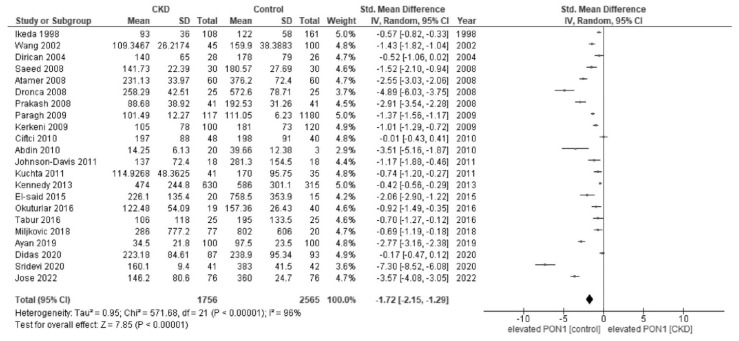
Forest plot of paraoxonase activity in all studies [26,27,28,29,30,31,32,33,34,35,36,37,38,39,40,41,42,44,46,47,48,49].

**Figure 3:**
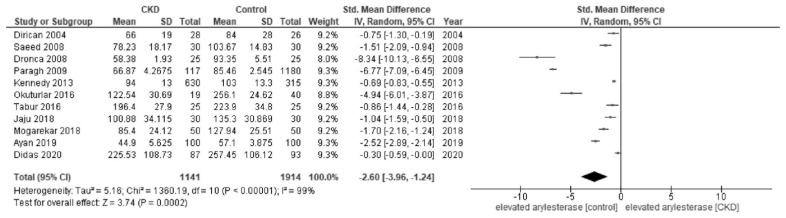
Forest plot of arylesterase activity in all studies [28,30,32,34,39,41,42,43,45,46,47].

In the meta-analysis, patients with CKD showed a lower activity of paraoxonase (SMD, −1.72; 95% CI, −2.15 to −1.29; I^2^ = 96%) than healthy controls (Figure 2). Patients with CKD showed a lower activity of arylesterase (SMD, −2.60; 95% CI, −3.96 to −1.24; I^2^ = 99%) than healthy controls (Figure 3). 

**Figure 4:**
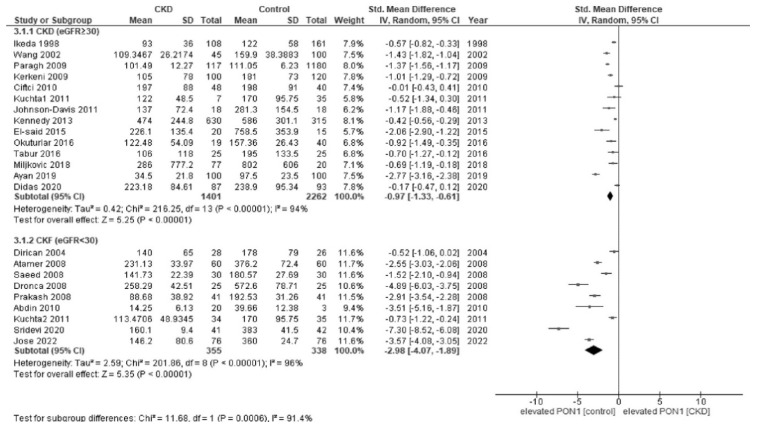
Forest plot of paraoxonase activity stratified by the CKD stages [26,27,28,29,30,31,32,33,34,35,36,37,38,39,40,41,42,44,46,47,48,49].

**Figure 5:**
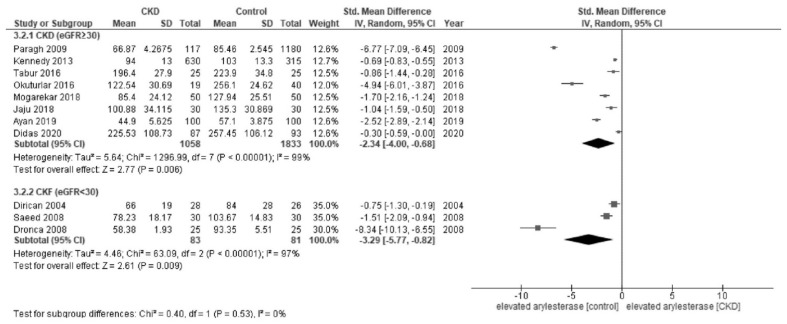
Forest plot of arylesterase activity stratified by the CKD stages [28,30,32,34,39,41,42,43,45,46,47].

In the subgroup analyses, there was a significant difference in paraoxonase activity by the CKD stages (*p* = 0.0006); patients with CKF (eGFR < 30 [6]) (SMD, −2.98; 95% CI, −4.07 to −1.89; I^2^ = 96%) had a lower activity of paraoxonase than those with non-CKF (eGFR ≥ 30) (SMD, −0.97; 95% CI, −1.33 to −0.61; I^2^ = 94%) (Figure 4). Regarding arylesterase activity, although there appeared to be similar trend to paraoxonase, the difference was not significant among the CKD stages (*p* = 0.53); patients with CKF (eGFR < 30) (SMD, −3.29; 95% CI, −5.77 to −1.89; I^2^ = 97%) had a relatively low but similar activity of arylesterase when compared to those with non-CKF (eGFR ≥ 30) (SMD, −2.34; 95% CI, −4.00 to −0.68; I^2^ = 99%) (Figure 5). The subgroup analyses by diabetes status and regions did not yield any relevant findings (Appendix C).

The sensitivity analysis showed similar trends in the main outcomes of all studies (Appendix D). We did not observe any publication bias (Appendix E).

## 4. Discussion

The main findings of our present study were that patients with CKD (without dialysis) had lower activities of PON1, both paraoxonase and arylesterase, than healthy controls. In addition, patients with CKF had a lower activity of PON1, paraoxonase in particular, than those with non-CKF. These results via meta-analysis are valuable to achieve a consensus that PON1 activity can be a relevant biomarker to evaluate CKD as well as its severity. 

The lower PON1 activity in patients with CKD would indicate an impairment of antioxidant defense by PON1 in this disease. Generally, patients with CKD have cardiometabolic disorders, such as a low level of HDL with a reduced antioxidant activity of HDL if accompanied by dysfunctionality of those HDL particles [6,7,8,9]. Uremic toxins (small and middle molecules), iron overload, angiotensin-2 elevation, and inflammatory cytokines induce excess oxidative stress in CKD [50]. Through the oxidative-antioxidative imbalance caused by these multiple conditions, an excess of oxidants inactivates PON1 and therefore can mechanistically explain the finding of lower PON1 activity observed in our present study. 

In our meta-analysis, when studies employed paraoxon as a substrate, a gradient of reduction of PON1 activity along the worsening of CKD stages became apparent. This may be explained by the lower levels of antioxidants found with advancing CKD status. On the other hand, this phenomenon showed the same trend, albeit it was not significant, when arylesterase activity was measured. As paraoxonase and arylesterase activities both measure generic PON1 activities [51,52], we should consider whether or not the difference was due to the small number of studies on arylesterase in comparison to those of paraoxonase in our present analysis [15]. On the other hand, the PON1 gene polymorphisms of 192 Gln/Arg and 55 Leu/Met in the amino-acid sequence partially alter the enzymatic activity of PON1 against paraoxon and not against arylesterase (i.e., 192 Gln/Arg QQ homozygotes have a lower activity of paraoxonase) [53,54,55] and we can assume such genetic effects on CKD stages. Thus, this point would merit further investigation, as the studies included in our present analysis did not explore such effects of specific polymorphisms. 

This study had several limitations. First, the range of PON1 activities was broad across studies. This is partly based on a lack of standardization of PON1 measurements. This must be overcome if PON1 activity is to be used routinely in clinical settings. Second, the impact of PON1 activity on clinical outcomes (e.g., cardiovascular events [56,57]) in patients with CKD was not investigated in the studies included in our present work. Third, although PON1 activity might be affected by lifestyles (e.g., diet [53,54]), this was not examined in the studies included in our present analysis. Fourth, although we conducted subgroup analyses for heterogeneity, the reasons for the apparent high heterogeneity remain unclear. Clarification of this issue is a future challenge. 

## 5. Conclusions

The present meta-analysis revealed that PON1 activity was low in patients with CKD (without dialysis), indicating that antioxidant defense by PON1 could be impaired in CKD. Lower PON1 activity levels were found in an advanced stage of CKD, as CKF, albeit this phenomenon might differ by PON1-species. Further studies are warranted to firmly establish PON1 activity as a relevant prognostic biomarker in CKD. 

## Figures and Tables

**Table 1:** Summary of the included articles on PON1 activities in patients with CKD.

First Author	Year [Ref. No.]	Country	Subject No.	Age	Unit	Activity in CKD	Activity in Healthy Controls	CKD/CKF Stages
**Paraoxonase**								
Ikeda	1998 [26]	Japan	169	57	U/L	93 ± 36.0	122.0 ± 58.0	CKD
Wang	2002 [27]	China	145	59	U/mL	109.3 ± 26.2	159.9 ± 38.3	CKD
Dirican	2004 [28]	Turkey	72	47	U/L	140.0 ± 65.0	178.0 ± 79.0	CKF
Atamer	2008 [29]	Turkey	60	53	U/L	231.1 ± 34.0	376.2 ± 72.4	CKF
Dronca	2008 [30]	Romania	50	51	U/L	258.3 ± 42.5	572.6 ± 78.7	CKF
Prakash	2008 [31]	India	89	53	U/L	88.7 ± 38.9	192.5 ± 31.3	CKF
Saeed	2008 [32]	Egypt	60	42	U/L	141.7 ± 22.4	180.6 ± 27.7	CKF
Kerkeni	2009 [33]	Tunisia	220	51	U/mL	105.0 ± 78.0	181.0 ± 73.0	CKD
Paragh	2009 [34]	Hungary	1297	48	U/L	101.5 ± 12.3	111.1 ± 6.2	CKF
Abdin	2010 [35]	Egypt	50	54	U/mL	14.3 ± 6.1	39.7 ± 12.4	CKF
Ciftci	2010 [36]	Turkey	88	31	U/L	197.0 ± 88.0	198.0 ± 91.0	CKD
Johnson-Davis	2011 [37]	USA	36	18–84	U/L	137.0 ± 72.4	281.3 ± 154.5	CKD
Kuchta	2011 [38]	Poland	76	58	U/L	114.9 ± 48.4	170.0 ± 95.8	CKD/CKF
Kennedy	2013 [39]	USA	945	69	U/L	474.0 ± 244.8	586.0 ± 301.1	CKD
EI-said	2015 [40]	Egypt	35	50	U/L	226.1 ± 135.4	758.5 ± 353.9	CKD
Okuturlar	2016 [41]	Turkey	59	55	U/L	122.5 ± 54.1	157.4 ± 26.4	CKD
Tabur	2016 [42]	Turkey	50	51	U/mL	106.0 ± 118.0	115.0 ± 101.0	CKD
Miljkovic	2018 [44]	Serbia	41	57	U/L	286.0 ± 777.2	802.0 ± 606.0	CKD
Ayan	2019 [46]	Turkey	200	55	U/L	34.5 ± 21.8	97.5 ± 23.5	CKD
Didas	2020 [47]	Thailand	180	68	U/L	223.2 ± 84.6	238.9 ± 95.3	CKD
Sridevi	2021 [48]	India	81	20–60	U/L	160.1 ± 9.4	383.0 ± 41.5	CKF
Jose	2022 [49]	India	152	51	U/L	146.2 ± 80.6	360.0 ± 24.7	CKF
**Arylesterase**								
Dirican	2004 [28]	Turkey	72	47	kU/L	66.0 ± 19.0	84.0 ± 28.0	CKF
Dronca	2008 [30]	Romania	50	51	U/L	58.4 ± 1.9	93.4 ± 5.5	CKF
Saeed	2008 [32]	Egypt	60	42	kU/L	78.2 ± 18.2	103.7 ± 14.8	CKF
Paragh	2009 [34]	Hungary	1297	47.6	U/mL	66.9 ± 4.3	85.5 ± 2.5	CKD
Kennedy	2013 [39]	USA	945	69	U/mL	94.0 ± 13.0	103.0 ± 13.3	CKD
Okuturlar	2016 [41]	Turkey	59	55	U/L	122.5 ± 30.7	256.1 ± 24.6	CKD
Tabur	2016 [42]	Turkey	50	51	U/mL	196.4 ± 27.9	223.9 ± 34.8	CKD
Jaju	2018 [43]	India	60	NR	NR	100.9 ± 34.2	135.3 ± 30.9	CKD
Mogarekar	2018 [45]	India	100	59	kU/L	85.4 ± 24.1	127.9 ± 25.5	CKD
Ayan	2019 [46]	Turkey	200	55	kU/L	44.9 ± 5.6	57.1 ± 3.9	CKD
Didas	2020 [47]	Thailand	87	68	kU/L	225.5 ± 108.7	257.5 ± 106.1	CKD

CKD, chronic kidney disease; CKF, chronic kidney failure; NR, not reported.

**Figure A1:**
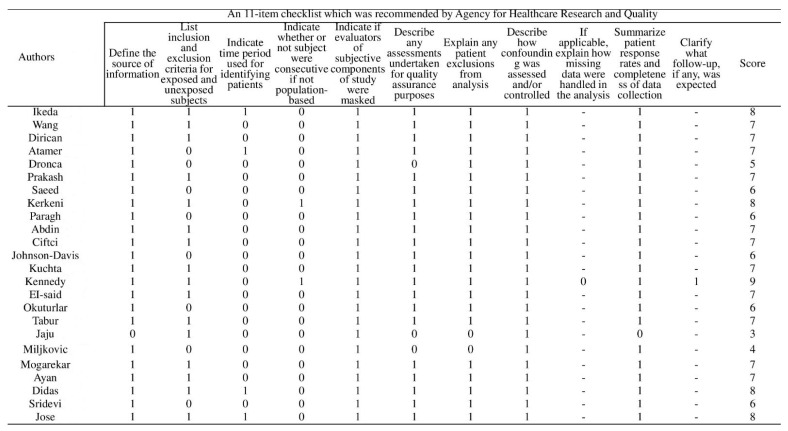
Details of risk of bias assessment [26,27,28,29,30,31,32,33,34,35,36,37,38,39,40,41,42,43,44,45,46,47,48,49].

**Figure A2:**
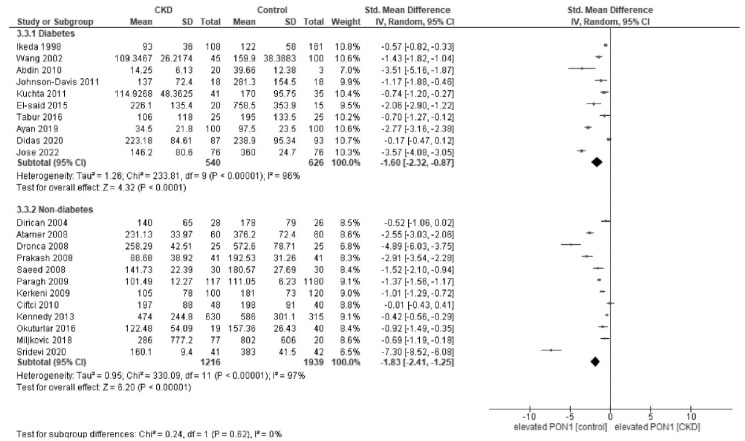
Forest plot of paraoxonase activity stratified by diabetes status [26,27,28,29,30,31,32,33,34,35,36,37,38,39,40,41,42,44,46,47,48,49].

**Figure A3:**
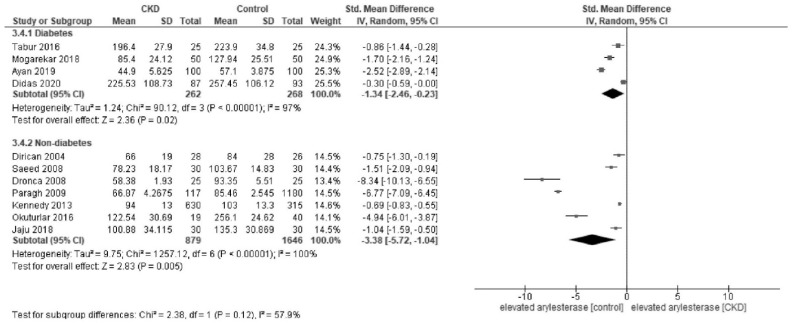
Forest plot of arylesterase activity stratified by diabetes status [28,30,32,34,39,41,42,43,45,46,47].

**Figure A4:**
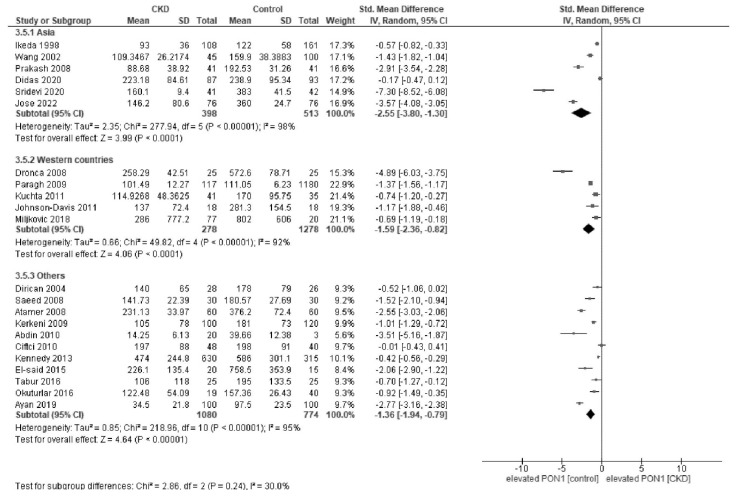
Forest plot of paraoxonase activity stratified by regions [26,27,28,29,30,31,32,33,34,35,36,37,38,39,40,41,42,44,46,47,48,49].

**Figure A5:**
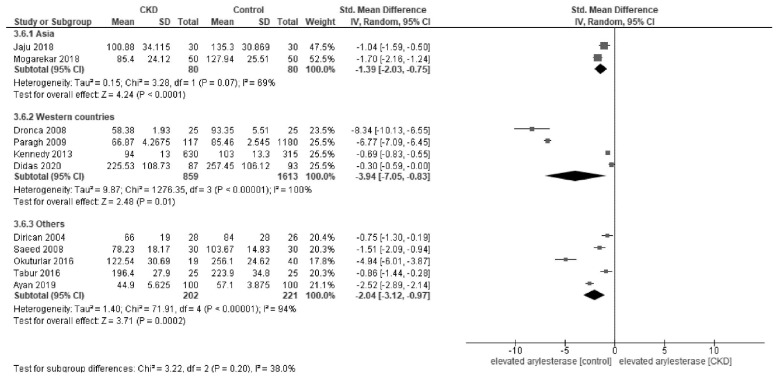
Forest plot of arylesterase activity stratified by regions [28,30,32,34,39,41,42,43,45,46,47].

**Figure A6:**
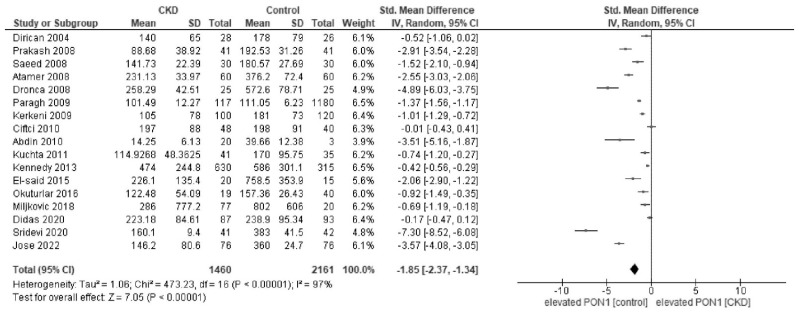
Forest plot of paraoxonase activity in the sensitivity analysis [26,27,28,29,30,31,32,33,34,35,36,37,38,39,40,41,42,44,46,47,48,49].

**Figure A7:**
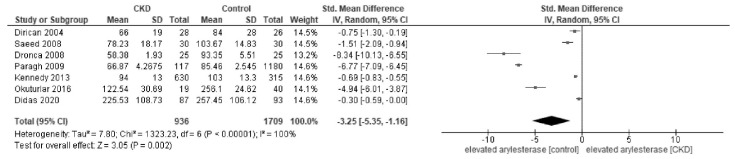
Forest plot of arylesterase activity in the sensitivity analysis [28,30,32,34,39,41,42,43,45,46,47].

**Figure A8:**
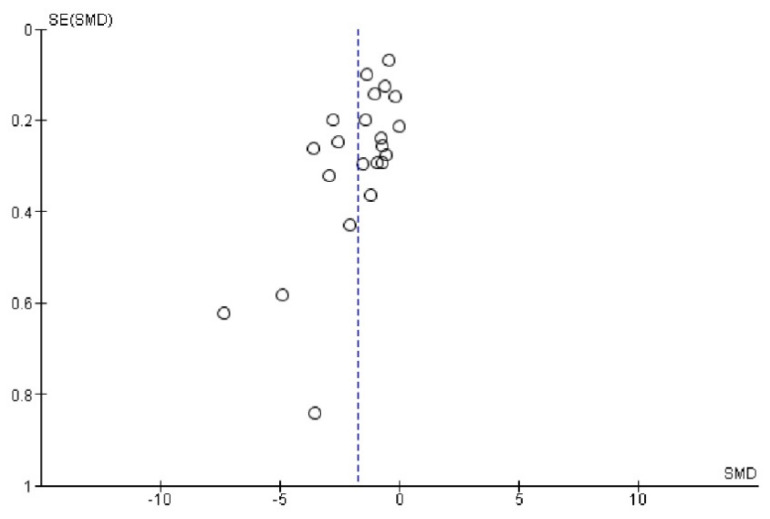
Funnel plot of paraoxonase activity (Egger test > 0.05).

**Figure A9:**
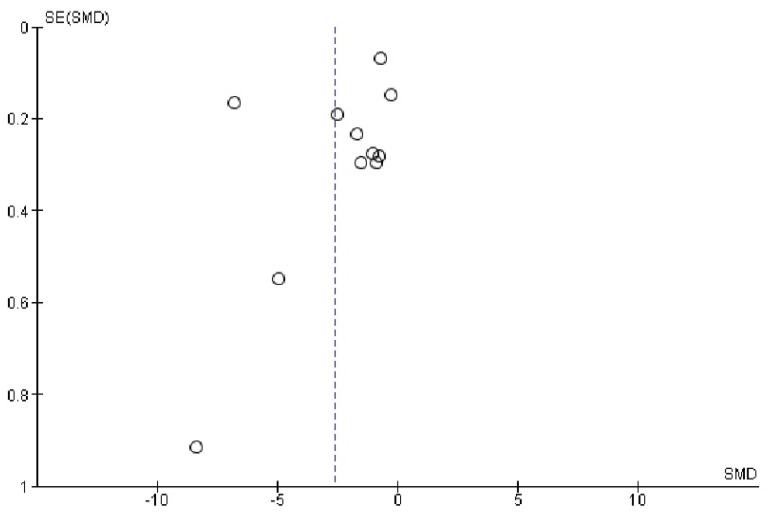
Funnel plot of arylesterase activity (Egger test > 0.05).

## Data Availability

All data relevant to the study are included in the article.

## Appendix A. Search Strategy

MEDLINE via PubMed

#1. “Aryldialkylphosphatase”[Mesh]

#2. “aryldialkylphosphatase”[tiab]

#3. “arylesterase”[tiab]

#4. “paraoxonase”[tiab]

#5. #1 OR #2 OR #3 OR #4

#6. “Renal Insufficiency”[Mesh]

#7. “Kidney Failure, Chronic”[Mesh]

#8. “Renal Insufficiency, Chronic”[Mesh]

#9. “Kidney Diseases”[Mesh]

#10. “Uremia”[Mesh]

#11. “end-stage renal”[tiab] OR “end-stage kidney”[tiab] OR “endstage renal”[tiab] OR “endstage kidney”[tiab]

#12. “ESRF”[tiab] OR “ESKF”[tiab] OR “ESRD”[tiab] OR “ESKD”[tiab]

#13. “chronic kidney”[tiab] OR “chronic renal”[tiab]

#14. “CKF”[tiab] OR “CKD”[tiab] OR “CRF”[tiab] OR “CRD”[tiab]

#15. “predialysis”[tiab] OR “predialysis”[tiab]

#16. #6 OR #7 OR #8 OR #9 OR #10 OR #11 OR #12 OR #13 OR #14 OR #15

#17. #5 AND #16

CENTRAL via Cochrane Library

#1. MeSH descriptor: [Aryldialkylphosphatase] explode all trees

#2. aryldialkylphosphatase:ti,ab

#3. arylesterase:ti,ab

#4. paraoxonase:ti,ab

#5. #1 OR #2 OR #3 OR #4

#6. MeSH descriptor: [Renal Insufficiency] explode all trees

#7. MeSH descriptor: [Kidney Failure, Chronic] explode all trees

#8. MeSH descriptor: [Renal Insufficiency, Chronic] explode all trees

#9. MeSH descriptor: [Kidney Diseases] explode all trees

#10. MeSH descriptor: [Uremia] explode all trees

#11. “end-stage renal”:ti,ab OR “end-stage kidney”:ti,ab OR “endstage renal”:ti,ab OR “endstage kidney”:ti,ab

#12. ESRF:ti,ab OR ESKF:ti,ab OR ESRD:ti,ab OR ESKD:ti,ab

#13. “chronic kidney”:ti,ab OR “chronic renal”:ti,ab

#14. CKF:ti,ab OR CKD:ti,ab OR CRF:ti,ab OR CRD:ti,ab

#15. predialysis:ti,ab OR predialysis:ti,ab

#16. #6 OR #7 OR #8 OR #9 OR #10 OR #11 OR #12 OR #13 OR #14 OR #15

#17. #5 AND #16

Embase via Proquest

S1 EMB.EXACT.EXPLODE(“aryldialkylphosphatase”)

S2 ab(aryldialkylphosphatase) OR ti(aryldialkylphosphatase)

S3 ab(arylesterase) OR ti(arylesterase)

S4 ab(paraoxonase) OR ti(paraoxonase)

S5 S1 OR S2 OR S3 OR S4

S6. EMB.EXACT.EXPLODE(“kidney failure”)

S7. EMB.EXACT.EXPLODE(“chronic kidney failure”)

S8. EMB.EXACT.EXPLODE(“chronic kidney failure”)

S9. EMB.EXACT.EXPLODE(“kidney disease”)

S10. EMB.EXACT.EXPLODE(“uremia”)

S11. ab(end-stage renal) OR ti(end-stage renal) OR ab(end-stage kidney) OR ti(end-stage kidney) OR ab(endstage renal) OR ti(endstage renal) OR ab(endstage kidney) OR ti(endstage kidney)

S12. ab(ESRF) OR ti(ESRF) OR ab(ESKF) OR ti(ESKF) OR ab(ESRD) OR ti(ESRD) OR ab(ESKD) OR ti(ESKD)

S13. ab(chronic kidney) OR ti(chronic kidney) OR ab(chronic renal) OR ti(chronic renal)

S14. ab(CKF) OR ti(CKF) OR ab(CKD) OR ti(CKD) OR ab(CRF) OR ti(CRF) OR ab(CRD) OR ti(CRD)

S15. ab(predialysis) OR ti(predialysis) OR ab(predialysis) OR ti(predialysis)

S16. S6 OR S7 OR S8 OR S9 OR S10 OR S11 OR S12 OR S13 OR S14 OR S15

S17. S5 AND S16

## References

[1] Matsushita K., Coresh J., Sang Y., Chalmers J., Fox C., Guallar E., Jafar T., Jassal S.K., Landman G.W.D., Muntner P. (2015). Estimated Glomerular Filtration Rate and Albuminuria for Prediction of Cardiovascular Outcomes: A Collaborative Meta-Analysis of Individual Participant Data. Lancet Diabetes Endocrinol..

[2] Webster A.C., Nagler E.V., Morton R.L., Masson P. (2017). Chronic Kidney Disease. Lancet.

[3] Cheung A.K., Chang T.I., Cushman W.C., Furth S.L., Hou F.F., Ix J.H., Knoll G.A., Muntner P., Pecoits-Filho R., Sarnak M.J. (2021). Kidney Disease: Improving Global Outcomes (KDIGO) Blood Pressure Work Group KDIGO 2021 Clinical Practice Guideline for the Management of Blood Pressure in Chronic Kidney Disease. Kidney Int..

[4] Hill N.R., Fatoba S.T., Oke J.L., Hirst J.A., O’Callaghan C.A., Lasserson D.S., Hobbs F.D.R. (2016). Global Prevalence of Chronic Kidney Disease—A Systematic Review and Meta-Analysis. PLoS ONE.

[5] Ene-Iordache B., Perico N., Bikbov B., Carminati S., Remuzzi A., Perna A., Islam N., Bravo R.F., Aleckovic-Halilovic M., Zou H. (2016). Chronic Kidney Disease and Cardiovascular Risk in Six Regions of the World (ISN-KDDC): A Cross-Sectional Study. Lancet Glob. Health.

[6] Isakova T., Nickolas T.L., Denburg M., Yarlagadda S., Weiner D.E., Gutiérrez O.M., Bansal V., Rosas S.E., Nigwekar S., Yee J. (2017). KDOQI US Commentary on the 2017 KDIGO Clinical Practice Guideline Update for the Diagnosis, Evaluation, Prevention, and Treatment of Chronic Kidney Disease-Mineral and Bone Disorder (CKD-MBD). Am. J. Kidney Dis..

[7] Matsushita K., Ballew S.H., Wang A.Y.-M., Kalyesubula R., Schaeffner E., Agarwal R. (2022). Epidemiology and Risk of Cardiovascular Disease in Populations with Chronic Kidney Disease. Nat. Rev. Nephrol..

[8] Ferro C.J., Mark P.B., Kanbay M., Sarafidis P., Heine G.H., Rossignol P., Massy Z.A., Mallamaci F., Valdivielso J.M., Malyszko J. (2018). Lipid Management in Patients with Chronic Kidney Disease. Nat. Rev. Nephrol..

[9] Grundy S.M., Stone N.J., Bailey A.L., Beam C., Birtcher K.K., Blumenthal R.S., Braun L.T., de Ferranti S., Faiella-Tommasino J., Forman D.E. (2019). 2018 AHA/ACC/AACVPR/AAPA/ABC/ACPM/ADA/AGS/APhA/ASPC/NLA/PCNA Guideline on the Management of Blood Cholesterol: A Report of the American College of Cardiology/American Heart Association Task Force on Clinical Practice Guidelines. Circulation.

[10] Noels H., Lehrke M., Vanholder R., Jankowski J. (2021). Lipoproteins and Fatty Acids in Chronic Kidney Disease: Molecular and Metabolic Alterations. Nat. Rev. Nephrol..

[11] Mackness M., Mackness B. (2013). Targeting Paraoxonase-1 in Atherosclerosis. Expert Opin. Ther. Targets.

[12] Vivekananthan D.P., Penn M.S., Sapp S.K., Hsu A., Topol E.J. (2003). Use of Antioxidant Vitamins for the Prevention of Cardiovascular Disease: Meta-Analysis of Randomised Trials. Lancet.

[13] Márquez A.B., Nazir S., van der Vorst E.P.C. (2020). High-Density Lipoprotein Modifications: A Pathological Consequence or Cause of Disease Progression?. Biomedicines.

[14] Soran H., Younis N.N., Charlton-Menys V., Durrington P. (2009). Variation in Paraoxonase-1 Activity and Atherosclerosis. Curr. Opin. Lipidol..

[15] Bhattacharyya T., Nicholls S.J., Topol E.J., Zhang R., Yang X., Schmitt D., Fu X., Shao M., Brennan D.M., Ellis S.G. (2008). Relationship of Paraoxonase 1 (PON1) Gene Polymorphisms and Functional Activity with Systemic Oxidative Stress and Cardiovascular Risk. JAMA.

[16] Ferretti G., Bacchetti T., Sahebkar A. (2015). Effect of Statin Therapy on Paraoxonase-1 Status: A Systematic Review and Meta-Analysis of 25 Clinical Trials. Prog. Lipid Res..

[17] Watanabe J., Kotani K., Gugliucci A. (2021). Paraoxonase 1 and Chronic Obstructive Pulmonary Disease: A Meta-Analysis. Antioxidants.

[18] Kotani K., Watanabe J., Miura K., Gugliucci A. (2021). Paraoxonase 1 and Non-Alcoholic Fatty Liver Disease: A Meta-Analysis. Molecules.

[19] Bassu S., Mangoni A.A., Argiolas D., Carru C., Pirina P., Fois A.G., Zinellu A. (2022). A Systematic Review and Meta-Analysis of Paraoxonase-1 Activity in Asthma. Clin. Exp. Med..

[20] Gugliucci A., Mehlhaff K., Kinugasa E., Ogata H., Hermo R., Schulze J., Kimura S. (2007). Paraoxonase-1 Concentrations in End-Stage Renal Disease Patients Increase after Hemodialysis: Correlation with Low Molecular AGE Adduct Clearance. Clin. Chim. Acta.

[21] Gugliucci A., Kinugasa E., Kotani K., Caccavello R., Kimura S. (2011). Serum Paraoxonase 1 (PON1) Lactonase Activity Is Lower in End-Stage Renal Disease Patients than in Healthy Control Subjects and Increases after Hemodialysis. Clin. Chem. Lab. Med..

[22] Gugliucci A., Kotani K., Kimura S. (2012). Paraoxonase 1 in Chronic Kidney Failure. J. Lipids.

[23] Page M.J., McKenzie J.E., Bossuyt P.M., Boutron I., Hoffmann T.C., Mulrow C.D., Shamseer L., Tetzlaff J.M., Akl E.A., Brennan S.E. (2021). The PRISMA 2020 Statement: An Updated Guideline for Reporting Systematic Reviews. BMJ.

[24] Viswanathan M., Ansari M.T., Berkman N.D., Chang S., Hartling L., McPheeters M., Santaguida P.L., Shamliyan T., Singh K., Tsertsvadze A. (2012). Assessing the Risk of Bias of Individual Studies in Systematic Reviews of Health Care Interventions. Agency for Healthcare Research and Quality Methods Guide for Comparative Effectiveness Reviews. https://effectivehealthcare.ahrq.gov/.

[25] Higgins J.P.T., Thomas J. (2022). Cochrane Handbook for Systematic Reviews of Interventions Version 6.3. https://training.cochrane.org/handbook/current.

[26] Ikeda Y., Suehiro T., Inoue M., Nakauchi Y., Morita T., Arii K., Ito H., Kumon Y., Hashimoto K. (1998). Serum Paraoxonase Activity and Its Relationship to Diabetic Complications in Patients with Non-Insulin-Dependent Diabetes Mellitus. Metabolism.

[27] Wang H., Deng H., Liu W. (2002). The effects of paraoxonase-1 and oxidized low density lipoprotein on nephropathy in type-2 diabetes mellitus. Zhonghua Nei Ke Za Zhi.

[28] Dirican M., Akca R., Sarandol E., Dilek K. (2004). Serum Paraoxonase Activity in Uremic Predialysis and Hemodialysis Patients. J. Nephrol..

[29] Atamer A., Kocyigit Y., Ecder S.A., Selek S., Ilhan N., Ecder T., Atamer Y. (2008). Effect of Oxidative Stress on Antioxidant Enzyme Activities, Homocysteine and Lipoproteins in Chronic Kidney Disease. J. Nephrol..

[30] Dronca M., Paşca S.P., Nemeş B., Vlase L., Vladutiu D. (2008). Serum Paraoxonase 1 Activities and Homocysteinemia in Hemodialysis Patients. Clin. Chem. Lab. Med..

[31] Prakash M., Shetty J.K., Rao L., Sharma S., Rodrigues A., Prabhu R. (2008). Serum Paraoxonase Activity and Protein Thiols in Chronic Renal Failure Patients. Indian J. Nephrol..

[32] Saeed S.A., Elsharkawy M., Elsaeed K., Fooda O. (2008). Paraoxonase-1 (PON1) Activity as a Risk Factor for Atherosclerosis in Chronic Renal Failure Patients. Hemodial. Int..

[33] Kerkeni M., Letaief A., Achour A., Miled A., Trivin F., Maaroufi K. (2009). Hyperhomocysteinemia, Paraoxonase Concentration and Cardiovascular Complications in Tunisian Patients with Nondiabetic Renal Disease. Clin. Biochem..

[34] Paragh G., Seres I., Harangi M., Pocsai Z., Asztalos L., Locsey L., Szeles G., Kardos L., Varga E., Karpati I. (2009). Discordance in Human Paraoxonase-1 Gene between Phenotypes and Genotypes in Chronic Kidney Disease. Nephron Clin. Pract..

[35] Abdin A.A., Hassanien M.A., Ibrahim E.A., El-Noeman S.E.-D.A.A. (2010). Modulating Effect of Atorvastatin on Paraoxonase 1 Activity in Type 2 Diabetic Egyptian Patients with or without Nephropathy. J. Diabetes Complicat..

[36] Ciftci H., Savas M., Yeni E., Verit A., Celik H., Oncel H. (2010). Serum Paraoxonase Activity in Patients with Low Glomerular Filtration Rates. Ren. Fail..

[37] Johnson-Davis K.L., Fernelius C., Eliason N.B., Wilson A., Beddhu S., Roberts W.L. (2011). Blood Enzymes and Oxidative Stress in Chronic Kidney Disease: A Cross Sectional Study. Ann. Clin. Lab. Sci..

[38] Kuchta A., Pacanis A., Kortas-Stempak B., Cwiklińska A., Ziętkiewicz M., Renke M., Rutkowski B. (2011). Estimation of Oxidative Stress Markers in Chronic Kidney Disease. Kidney Blood Press. Res..

[39] Kennedy D.J., Tang W.H.W., Fan Y., Wu Y., Mann S., Pepoy M., Hazen S.L. (2013). Diminished Antioxidant Activity of High-Density Lipoprotein-Associated Proteins in Chronic Kidney Disease. J. Am. Heart Assoc..

[40] El-said N.H., Nasr-Allah M.M., Sadik N.A., Sharaf S.A. (2015). Paraoxonase-1 Activity in Type 2 Diabetes Mellitus with and without Nephropathy. Egypt. J. Intern. Med..

[41] Okuturlar Y., Akalin N., Kaptanogullari O.H., Guner N.T., Yilmaz D., Gedikbasi A., Soyluk O., Mert M., Serin S.O., Kocoglu H. (2016). Comparison of Serum Paraoxonase and Arylesterase Activities between Iron Deficiency Anemia Patients and Chronic Kidney Disease Patients with Anemia. Ren. Fail..

[42] Tabur S., Korkmaz H., Eren M.A., Oğuz E., Sabuncu T., Kul S., Aksoy N. (2016). Can Visfatin Be Considered as a Diagnostic Marker for Diabetic Nephropathy?. Turk. J. Endocrinol. Metab..

[43] Jaju J.B., Dhabe M.G. (2018). Study of Serum Carbamylated Total Protein and Paraoxonase 1 Levels in Chronic Kidney Disease. Indian J. Clin. Biochem..

[44] Miljkovic M., Stefanovic A., Simic-Ogrizovic S., Vekic J., Bogavac-Stanojevic N., Cerne D., Kocbek P., Marc J., Jelic-Ivanovic Z., Spasojevic-Kalimanovska V. (2018). Association of Dyslipidemia, Oxidative Stress, and Inflammation With Redox Status in VLDL, LDL, and HDL Lipoproteins in Patients With Renal Disease. Angiology.

[45] Mogarekar M.R., Dhabe M.G., Palmate M.M. (2018). Paraoxonase 1 Activity and Its Polymorphism in Type 2 Diabetic Nephropathy. Turk. J. Endocrinol. Metab..

[46] Ayan D., Şeneş M., Çaycı A.B., Söylemez S., Eren N., Altuntaş Y., Öztürk F.Y. (2019). Evaluation of Paraoxonase, Arylesterase, and Homocysteine Thiolactonase Activities in Patients with Diabetes and Incipient Diabetes Nephropathy. J. Med. Biochem..

[47] Didas N., Thitisopee W., Porntadavity S., Jeenduang N. (2020). Arylesterase Activity but Not PCSK9 Levels Is Associated with Chronic Kidney Disease in Type 2 Diabetes. Int. Urol. Nephrol..

[48] Sridevi C., Sowjanya U., Selvi V.S.K., Rajakumari D.M.M., Babu K. (2021). Serum Paraoxonase with HDL-C as a Predictor of Atherosclerosis in Patients of Chronic Kidney Disease. Biomedicine.

[49] Jose S., Kc R., Chandran S. (2022). Oxidative Stress in Patients with Diabetic Nephropathy. Natl. J. Physiol. Pharm. Pharmacol..

[50] Ebert T., Neytchev O., Witasp A., Kublickiene K., Stenvinkel P., Shiels P.G. (2021). Inflammation and Oxidative Stress in Chronic Kidney Disease and Dialysis Patients. Antioxid. Redox Signal..

[51] Aviram M., Rosenblat M., Billecke S., Erogul J., Sorenson R., Bisgaier C.L., Newton R.S., La Du B. (1999). Human Serum Paraoxonase (PON 1) Is Inactivated by Oxidized Low Density Lipoprotein and Preserved by Antioxidants. Free Radic. Biol. Med..

[52] Camps J., Marsillach J., Joven J. (2009). The Paraoxonases: Role in Human Diseases and Methodological Difficulties in Measurement. Crit. Rev. Clin. Lab. Sci..

[53] Yu Z.-Y., Chen L.-S., Zhang L.-C., Zhou T.-B. (2012). Meta-Analysis of the Relationship between ACE I/D Gene Polymorphism and End-Stage Renal Disease in Patients with Diabetic Nephropathy. Nephrology.

[54] Tziastoudi M., Cholevas C., Theoharides T.C., Stefanidis I. (2021). Meta-Analysis and Bioinformatics Detection of Susceptibility Genes in Diabetic Nephropathy. Int. J. Mol. Sci..

[55] Gbandjaba N.Y., Ghalim N., Hassar M., Berrougui H., Labrazi H., Taki H., Saile R., Khalil A. (2012). Paraoxonase Activity in Healthy, Diabetic, and Hemodialysis Patients. Clin. Biochem..

[56] Dirican M., Sarandol E., Serdar Z., Ocak N., Dilek K. (2007). Oxidative Status and Prevalent Cardiovascular Disease in Patients with Chronic Renal Failure Treated by Hemodialysis. Clin. Nephrol..

[57] Suematsu Y., Goto M., Park C., Nunes A.C.F., Jing W., Streja E., Rhee C.M., Cruz S., Kashyap M.L., Vaziri N.D. (2019). Association of Serum Paraoxonase/Arylesterase Activity with All-Cause Mortality in Maintenance Hemodialysis Patients. J. Clin. Endocrinol. Metab..

